# The Potential of Theophylline and Pentoxifylline in Sperm Optimization and Its Intracytoplasmic Sperm Injection Outcomes

**DOI:** 10.7759/cureus.48192

**Published:** 2023-11-02

**Authors:** Bhavika Gala, Ankit Badge, Pranita Bawaskar, Ujwal Gajbe, Brij Raj Singh, Mangesh Kohale

**Affiliations:** 1 Clinical Embryology, Datta Meghe Medical College, Datta Meghe Institute of Higher Education & Research (Deemed to be University), Nagpur, IND; 2 Microbiology, Datta Meghe Medical College, Datta Meghe Institute of Higher Education & Research (Deemed to be University), Nagpur, IND; 3 Anatomy, Datta Meghe Medical College, Datta Meghe Institute of Higher Education & Research (Deemed to be University), Nagpur, IND; 4 Pathology, Datta Meghe Medical College, Datta Meghe Institute of Higher Education & Research (Deemed to be University), Nagpur, IND

**Keywords:** cyclic adenosine monophosphate, artificial sperm activation, phosphodiesterase inhibitors, pentoxifylline, theophylline

## Abstract

Sperm motility is an essential selection criteria by embryologists at the time of intracytoplasmic sperm injection (ICSI). One method of testing sperm viability is to induce sperm motility by increasing cyclic adenosine monophosphate (cAMP) levels by treating a semen sample with phosphodiesterase inhibitors (PDEIs), such as theophylline and pentoxifylline. It explores the implications of PDEI in medical care, reflecting on its effects in clinical settings and recognizing potential topics for future exploration. This analysis revealed that by incorporating stimulants that activate movements, the time it took to single out sperms was markedly reduced, and consequently, the sperms were safeguarded from a prolonged period of oxidative stress. Furthermore, theophylline was found to advance sperm motility, consequently resulting in several initially immobile spermatozoa displaying rapid progressive motility. Higher fertilization rate, cleavage rate, good quality embryos (grade I), and higher biochemical and clinical pregnancy rates were found with artificial sperm activation (ASA) using pentoxifylline and theophylline. This review emphasizes the need for more research to evaluate the drug's long-term safety and investigate the effects of theophylline and pentoxifylline on postfertilization parameters, such as embryo development, implantation, and pregnancy outcomes. These areas of investigation are important for understanding the complete impact of these agents and to ensure their safe and effective implementation in clinical practice.

## Introduction and background

Infertility is failing to conceive after one year of regular unprotected coitus. Infertility is observed in 15% of married couples, and 40% of infertility has been reported to be caused by men, 40% by women, and the remaining 20% being attributed to both sexes [1,2]. A major factor contributing to male infertility is the immobility of sperm [3]. When deciding on the most suitable sperm for intracytoplasmic sperm injection (ICSI), several approaches have been taken, such as the hypo-osmotic swelling (HOS) test; the inclusion of pentoxifylline, theophylline, or papaverine; the use of laser-assisted selection; the assessment of the flexibility of the sperm tail; and the utilization of the platelet activation factor (PAF) [4]. Chemical elements, such as caffeine and other methylxanthines, relaxin, 2-deoxyadenosine, and kallikrein, have been administered to test subjects orally, but most of them have been administered at the site [5]. Methylxanthine derivatives have been shown to increase the motility of ejaculated sperm. These substances inhibit the cyclic adenosine 3'5' monophosphate (cAMP) phosphodiesterase enzyme, producing a higher concentration of cAMP within the cell [6]. The substances theophylline and pentoxifylline, which are 3'5'-nucleotidase phosphodiesterase inhibitors based on methylxanthine, raise the level of cAMP within the cell by hindering its degradation [7,8]. The rise in cAMP levels will spark a rise in oxygen consumption and metabolic activity that creates energy, which will increase sperm motility and begin the capacitation process [9]. Patients with varicocele have been taking pentoxifylline orally, which has been shown to positively affect their seminal characteristics. In other cases, it has been used to increase the capacity of samples with poor motility to fertilize [10,11]. Pentoxifylline promotes the penetration of cervical sperm mucus in addition to sperm motility and plasmalemma integrity [12].

The purpose of this review is to analyze the impact of phosphodiesterase inhibitors, theophylline, and pentoxifylline, on sperm motility and to review advancements in sperm selection.

## Review

Methods

The databases used in this study include PubMed and Google Scholar. The search terms were "theophylline," "pentoxifylline," "cAMP," and "phosphodiesterase inhibitors” carried out in the databases. Only articles in the English language were included in the review. This review surveyed studies exploring the effects of sperm motility-enhancing drugs on ICSI.

Article Screened

**Figure 1:**
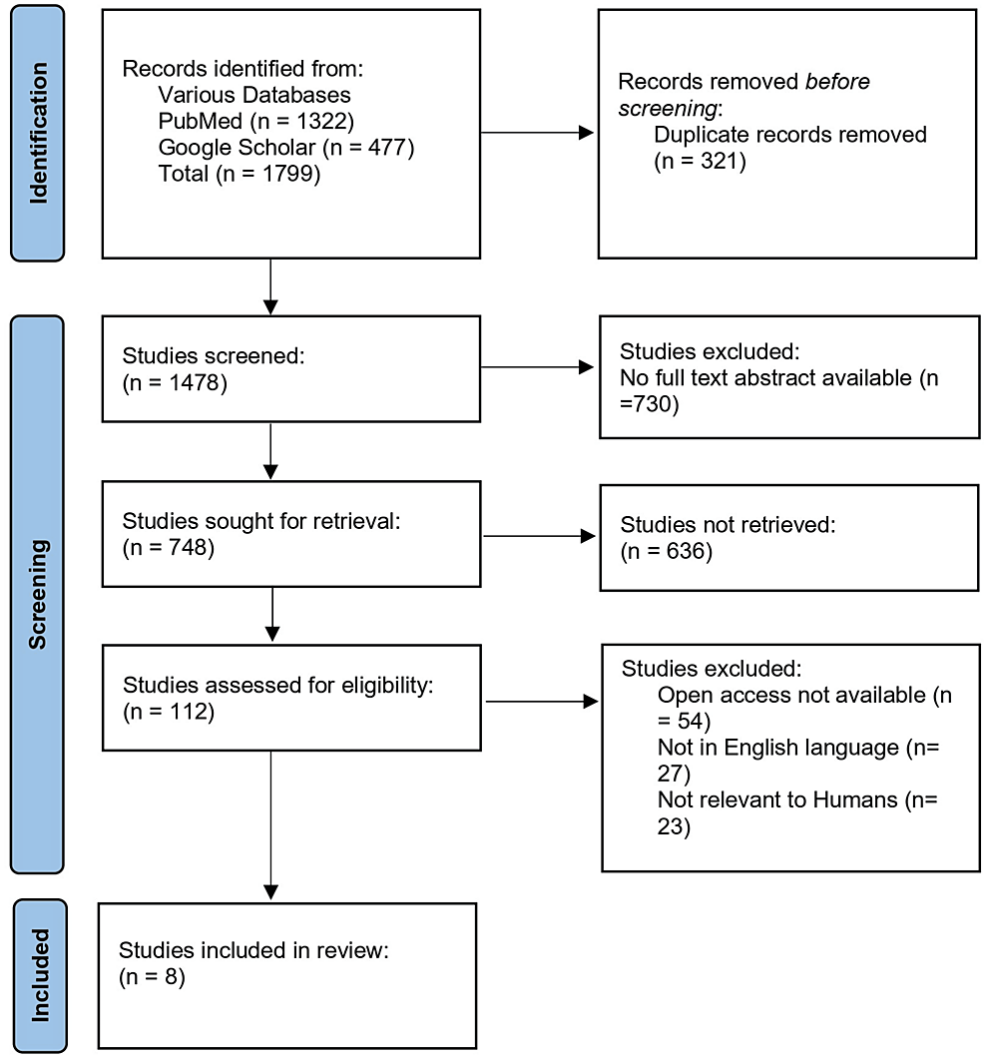
PRISMA flow chart n: number of studies, PRISMA: Preferred Reporting Items for Systematic Reviews and Meta-Analyses

After conducting the initial search, we identified 1,799 articles in the searched databases. We excluded duplicates (n=321) and completed an initial screening of titles and abstracts, excluding 730 articles. After the full-text screening of the remaining 748 articles, we excluded 636 articles because they did not meet the inclusion criteria. Then, we excluded 104 articles that were not available in open access, not available in the English language, and not relevant to humans. Consequently, the final review included a total of eight articles published from 2011 to 2023 (Figure 1).

Research studies on theophylline and pentoxifylline for sperm optimization are discussed in Table 1.

**Table 1:** Studies included in the review TESE: testicular sperm extraction, DNA: deoxyribonucleic acid, FNA: fine needle aspiration, MESA: microsurgical epididymal sperm aspiration, M-TESE: microsurgical testicular sperm extraction

Sr. No.	Author	Year	Drug	Participant	Findings
1.	Aydos et al. [13]	2022	Pentoxifylline	Non-obstructive azoospermia patients	Improved fertilization rates. No significant difference in pregnancy and live birth rates.
2.	Ebner et al. [14]	2011	Theophylline	Frozen-thawed TESE sample in azoospermia patients	Improved sperm motility. The theophylline-treated group had a shorter time for selecting sperm, higher rates of fertilization and blastulation, and more successful pregnancies.
3.	Ebner et al. [7]	2014	Theophylline	Retrograde ejaculation and total asthenozoospermia patient	Improved progressive motility of sperm. Successful fertilization and transfer of a single embryo achieved a healthy live birth.
4.	Unsal et al. [1]	2016	Pentoxifylline	Male patients with unknown cause of infertility	A notable rise in DNA damage observed. DNA damage was related to sperm motility, suggesting that external factors can affect sperm DNA stability.
5.	Mossa et al. [15]	2017	Theophylline	Non-obstructive azoospermia patients	No significant alteration in sperm count and morphology after using theophylline solution. A considerably larger number of high-grade embryos. A higher fertilization rate, implantation rate, biochemical pregnancy rate, and clinical pregnancy rate obtained.
6.	Javed et al. [4]	2017	Pentoxifylline and theophylline	Normal ejaculates, sperm retrieved surgically by FNA, MESA, M-TESE, and testicular sperm extraction TESE samples	Both pentoxifylline and theophylline worked in approximately 90% of cases. No significant difference in biochemical and clinical pregnancies. The pentoxifylline-treated group had a slightly higher fertilization rate but a notably higher incidence of miscarriages. Theophylline-treated cases had more successful deliveries of healthy babies.
7.	Sandy-Monroy et al. [10]	2019	Theophylline	Testicular sperm. Ejaculate with a concentration of less than 5 million/ml	Decline in clinical pregnancy loss and a slightly higher rate of live births. No records of perinatal mortality, congenital malformations, or need for intensive treatment in infants.
8.	Gandhi et al. [2]	2021	Theophylline	TESA samples	Rise in fertilization rate, cleavage rate with better quality embryos, and a higher rate of clinical pregnancy.

Out of the eight articles included, three studies [7,13,14] showed that the progressive motility of the sperm has improved, resulting in higher rates of successful fertilization. One study [1] noted a rise in DNA damage, which affected sperm motility and DNA stability. A higher fertilization rate, implantation rate, biochemical pregnancy rate, clinical pregnancy rate, and better quality embryos were obtained in two studies [2,15]. One study [4] compared the use of pentoxifylline and theophylline. Higher rates of live births and low rates of miscarriage were observed in other studies [10].

Mechanism of action of sperm motility enhancers

**Figure 2:**
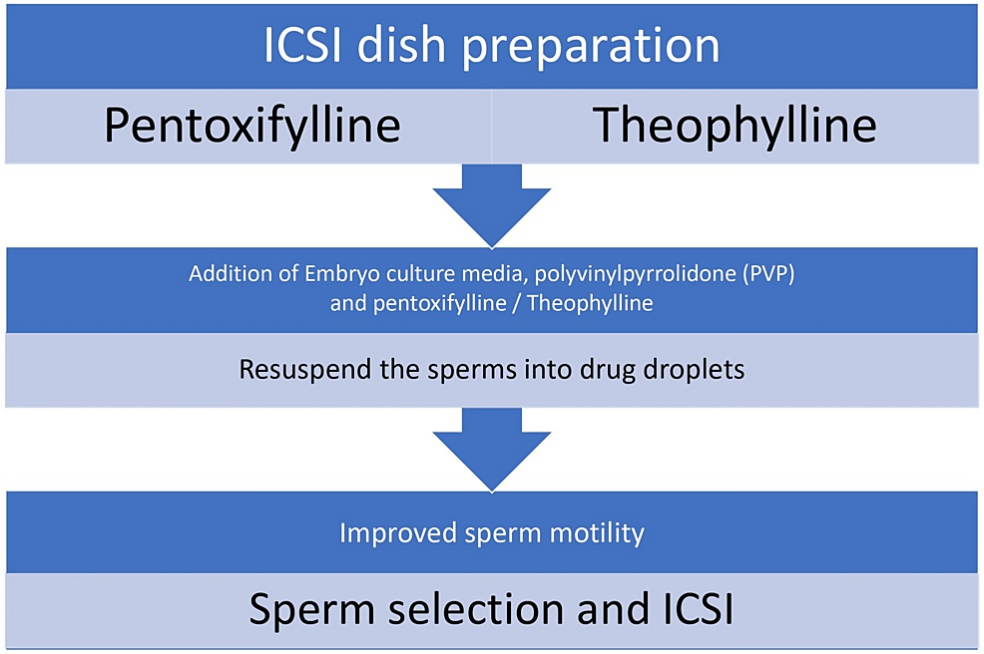
Method for using sperm motility enhancers. Source:  [10,16] ICSI: intracytoplasmic sperm injection

There are two approaches in handling sperm samples with pentoxifylline: adding a dose of pentoxifylline solution to the droplets of an ICSI dish and incorporating a particular amount of pentoxifylline concentrate into the resuspended sperm pellet. Both approaches have been found to improve sperm motility and increase the chances of successful fertilization. The first method allows for a direct exposure of the sperm to pentoxifylline, promoting immediate enhancement of motility [16]. Upon coming into contact with theophylline, sperm cells speed up, their heads drift more to the side, and their flagellar beat gains strength, increasing energy expenditure. This increased energy expenditure allows the sperm cells to swim faster and more vigorously toward the egg, increasing their chances of successful fertilization. Theophylline, a methylxanthine compound, acts as a phosphodiesterase inhibitor, preventing the breakdown of cAMP within the sperm cells. It is not known whether the effects of this can persist for several hours after exposure as a result of persistent inhibition of phosphodiesterase, high levels of cAMP, or any other cAMP-independent mechanisms [10]. Theophylline or methylxanthine has three primary ways of influencing cells: it transfers calcium from the cell to the outside; stops phosphodiesterase activity, resulting in an increased presence of cyclic AMP inside the cell; and blocks the action of adenosine receptors. This leads to bronchodilation and relaxation of smooth muscles, making it a commonly used medication for asthma and chronic obstructive pulmonary disease (COPD). In addition, theophylline has been found to have anti-inflammatory effects, reducing the production of cytokines and chemokines in airway cells [17,18]. The method for using sperm motility enhancers is shown in Figure 2.

Advantages of using theophylline

Theophylline improved the percentage of progressively motile sperm in retrograde ejaculation and total asthenozoospermia patients [7]. Using theophylline reduces the time it takes embryologists to select the best sperm during ICSI [2]. Being an oxygen radical scavenger, theophylline reduces lipid peroxidation, preventing oxidative stress that causes DNA damage and thus protecting the sperm membrane [15]. A higher fertilization rate and cleavage rate were obtained after ICSI [2]. An increased number of good quality embryos were developed after sperm was treated with theophylline [14]. theophylline-treated sperm resulted in higher biochemical pregnancies and clinical pregnancies [2,4,14]. A healthy live birth was achieved in the cases of retrograde ejaculation and total asthenozoospermia patients [7]. Low rate of miscarriage and a slightly higher rate of live births resulted after using theophylline [19]. There are no records of perinatal mortality, congenital malformations, or need for intensive treatment in infants with theophylline [19]. The short incubation time increases the capacitation process [9]. Theophylline is beneficial for fresh ejaculate, testicular sperm extraction (TESE) samples, testicular epididymal sperm aspiration (TESA) samples, surgically retrieved sperm by fine needle aspiration (FNA), microsurgical epididymal sperm aspiration (MESA), and microsurgical testicular sperm extraction (Micro-TESE) [4]. It is beneficial in the cases of nonobstructive azoospermia, male patients with unknown causes of infertility, retrograde ejaculation, and total asthenozoospermia patients [7,13]. No disadvantages were reported.

Advantages of using pentoxifylline

Pentoxifylline improved the percent sperm motility of grades I, II, and III and resulted in a good percentage of morphologically normal sperm. Pentoxifylline can be an effective treatment for male infertility, as it not only enhances sperm motility but also improves the overall quality of sperm. These findings are promising for couples struggling to conceive and may offer a viable solution to improve their chances of a successful pregnancy [20,21]. It stimulates the acrosome reaction in sperm, which is essential for sperm fertilization as it allows the sperm to penetrate and fuse with the egg. By enhancing the acrosome reaction, pentoxifylline increases the chances of successful fertilization and subsequent pregnancy [20]. Pentoxifylline promotes the penetration of cervical sperm mucus penetration in addition to sperm motility and integrity of the plasmalemma. This improves the chances of fertilization by facilitating the movement of sperm through the cervix and into the uterus. Furthermore, it helps to maintain the structural integrity of the outer membrane, ensuring its viability and ability to reach the egg [12]. Pentoxifylline can also reduce incubation and sperm selection time for ICSI, enhances several aspects of sperm activity, and is helpful in the ICSI-assisted reproductive procedure [22]. Pentoxifylline improves the rates of in vitro fertilization (IVF) methods and increases the number of high-quality embryos for successful implantation and pregnancy [13].

Disadvantages of using pentoxifylline

Pentoxifylline causes increased DNA damage, affecting sperm motility and sperm DNA stability [1]. There is no significant alteration in sperm count and morphology [15]. There is a higher incidence of miscarriages with pentoxifylline [4]. Pentoxifylline has been reported to have a detrimental effect on oocyte quality and its functioning and has also been found to be embryotoxic [23]. Long-term incubation can cause morphological alterations, retardation, or death of an embryo [24]. Malformations in the 10th revision of the International Classification of diseases 10th revision (ICD10) affecting the musculoskeletal system, eye, and intestine were recorded after birth [11].

Advancements in sperm selection

Embryologists now have approaches to identifying live sperm for ICSI from specimens that have exclusively immotile sperm. One potential method is to assess the flexibility of the sperm tail with an ICSI pipette. It is not known whether sperm possessing a flexible tail also has a functional nucleus. Another approach frequently employed is the HOS test, which examines the functionality of the sperm’s plasma membrane. When active sperm are placed in a hypo-osmotic solution, it will cause curling of the tail tip [25,26]. The preparation of a hypo-osmotic solution is simple and involves combining sperm wash medium and sterile deionized water in equal proportions. It is possible to cause the tail of the sperm to curl by administering a single shot of diode laser at the tip of the sperm's tail. However, this technique is dependent on the effective operation of the sperm's tail. The most reliable way is to partially recover sperm movement using a sperm motility enhancer [4]. Theophylline and pentoxifylline hold great promise in reproductive medicine by optimizing the outcomes of IVF. These agents improve sperm motility, capacitation, and fertilization rates and suggest their potential for managing male factor infertility. Furthermore, the reduction in sperm DNA damage by pentoxifylline highlights its possible role in reducing genetic abnormalities in offspring conceived through assisted reproduction techniques. Although pentoxifylline is used to improve motility, its application is not commonly approved due to the potential damage it could cause to oocytes, embryos, and newborn babies [27].

## Conclusions

Sperm motility enhancers do not appear to increase the chance of adverse obstetric and perinatal outcomes in a child. Theophylline is safer than pentoxifylline in identifying viable spermatozoa and has better live birth rates. Theophylline did not show a significant increase in adverse effects in pregnancy or the newborn. Furthermore, it was associated with improved sperm movement and higher success rates in achieving a live birth. These findings suggest that theophylline may be a more favorable option to enhance sperm motility without compromising obstetric and perinatal outcomes. Furthermore, a thorough and carefully planned research is necessary, such as preimplantation genetic diagnosis, genetic testing of the aborted fetus, and identification of major and minor congenital disabilities caused by the potential embryotoxicity of IVF treatment with these medications. In addition, long-term follow-up studies should be conducted to assess the possible impact of these medications on the overall health and development of children conceived through IVF. It is crucial to gather comprehensive data on any possible genetic abnormalities or adverse effects that may arise from these medications, ensuring the safety and well-being of future generations.
